# Evaluation of the Potential of *Lactobacillus paracasei* Adjuncts for Flavor Compounds Development and Diversification in Short-Aged Cheddar Cheese

**DOI:** 10.3389/fmicb.2018.01506

**Published:** 2018-07-05

**Authors:** Ewelina Stefanovic, Kieran N. Kilcawley, Clara Roces, Mary C. Rea, Maurice O'Sullivan, Jeremiah J. Sheehan, Olivia McAuliffe

**Affiliations:** ^1^Department of Food Biosciences, Teagasc Food Research Centre, Fermoy, Ireland; ^2^APC Microbiome Ireland, University College Cork, Cork, Ireland; ^3^School of Food and Nutritional Sciences, University College Cork, Cork, Ireland

**Keywords:** *Lactobacillus paracasei*, dairy, cheese flavor, diversification, volatiles

## Abstract

The non-starter microbiota of Cheddar cheese mostly comprises mesophilic lactobacilli, such as *Lactobacillus casei, Lactobacillus paracasei, Lactobacillus rhamnosus*, and *Lactobacillus plantarum*. These bacteria are recognized for their potential to improve Cheddar cheese flavor when used as adjunct cultures. In this study, three strains of *L. paracasei* (DPC2071, DPC4206, and DPC4536) were evaluated for their contribution to the enhancement and diversification of flavor in short-aged Cheddar cheese. The strains were selected based on their previously determined genomic diversity, variability in proteolytic enzyme activities and metabolic capability in cheese model systems. The addition of adjunct cultures did not affect the gross composition or levels of lipolysis of the cheeses. The levels of free amino acids (FAA) in cheeses showed a significant increase after 28 days of ripening. However, the concentrations of individual amino acids in the cheeses did not significantly differ except for some amino acids (aspartic acid, threonine, serine, and tryptophan) at Day 14. Volatile profile analysis revealed that the main compounds that differentiated the cheeses were of lipid origin, such as long chain aldehydes, acids, ketones, and lactones. This study demonstrated that the adjunct *L. paracasei* strains contributed to the development and diversification of compounds related to flavor in short-aged Cheddar cheeses.

## Introduction

The production of cheese globally increases year-on-year with an annual production of over 22 million tons (www.dairy.ahdb.org, report from February 27th, 2017). With such a high market demand, the dairy industry is challenged by increasing consumer requirements for products of novel flavor. Thus, the industry is seeking a means of enhancement and diversification of cheese flavor (Gobbetti et al., 2007).

Although general chemical composition of cheese has an influence on flavor development (Lynch et al., 1999), it is the metabolic activity driven by the cheese microbiota during ripening that represents the major driving force of flavor development (El Soda et al., 2000; Yvon, 2006). Proteolytic reactions that occur during cheese manufacture and ripening are seen as major contributors to texture and flavor development (McSweeney and Sousa, 2000). The main protein of milk is casein, and its degradation by rennet and intrinsic milk proteinases releases large peptides (primary proteolysis), that are further metabolized by proteinases of starter and non-starter (or adjunct) bacteria to release small peptides (Sousa et al., 2001). Subsequently, bacterial peptidases release free amino acids (FAA) (secondary proteolysis), which contribute directly to the cheese flavor, but also indirectly through their metabolism by microbial amino acid converting enzymes, which is considered to be one of the main flavor pathways (Ardo, 2006; Yvon, 2006). In addition to proteolysis, other pathways such as lipolysis and glycolysis also contribute to the cheese flavor. In the glycolytic/citrate pathway, pyruvate represents the central metabolite occurring from lactose or citrate metabolism, and it is further degraded to acetaldehyde, ethanol, diacetyl, and acetoin, all of which are important cheese flavor contributors (Marilley and Casey, 2004). The lipolytic pathways include a complex network of reactions, in which numerous long chain alcohols, acids, methyl-ketones and lactones are produced with various aroma notes (Collins et al., 2003). Since microorganisms present in cheese during the ripening differ in their metabolic abilities, the alteration of the microbial populations in the cheese represents a potential tool for flavor diversification (Van Hoorde et al., 2010).

The microbiota of Cheddar cheese comprises the starter lactic acid bacteria (SLAB) and non-starter lactic acid bacteria (NSLAB). SLAB acidify the milk during fermentation (El Soda et al., 2000), but they also contribute to the flavor development due to their metabolic activity (Wouters et al., 2002; Kieronczyk et al., 2003). NSLAB represent the endogenous secondary flora, and these organisms dominate the later stages of Cheddar cheese ripening (Wouters et al., 2002; Burns et al., 2012). The NSLAB population of Cheddar cheese includes homo-and heterofermentative mesophilic lactobacilli: *Lactobacillus casei, Lactobacillus paracasei, Lactobacillus rhamnosus, Lactobacillus plantarum*, and *Lactobacillus brevis* (Fitzsimons et al., 1999; Gobbetti et al., 2015). These bacteria show adaptability to environments with limited amounts of nutrients which occur in the later stages of Cheddar cheese ripening (Hussain et al., 2009). In this environment, they use mainly peptides and amino acids as nitrogen and energy sources, since residual lactose is present at low levels (Settanni and Moschetti, 2010). Additionally, nucleic acids, and sugars from glycoproteins and glycolipids of the lysed starters act as the potential substrates for NSLAB (Steele et al., 2006).

NSLAB have a prominent role in cheese flavor development (Crow et al., 2002), as cheeses made in aseptic conditions with starter bacteria developed poor flavor profiles (Wijesundera et al., 1997). Additionally, cheeses made with raw milk, containing higher levels of NSLAB than pasteurized milk, develop stronger flavor (Fox et al., 1998). NSLAB contribute to the intensification of flavor and increased overall acceptability of the cheese mainly through secondary proteolysis and the metabolism of FAA (McSweeney and Fox, 1997; Lynch et al., 1999; Di Cagno et al., 2006; Milesi et al., 2009). However, in some instances, non-starter flora can contribute to the formation of off- flavors, especially in the later phases of ripening (Crow et al., 2001; Antonsson et al., 2003; Gobbetti et al., 2015).

Because of their generally positive effect on cheese flavor development, members of the mesophilic lactobacilli are often deliberately added to cheese milk as adjunct cultures during industrial production. Apart from the direct impact on flavor development, they expedite the ripening and help to control adventitious microflora (Milesi et al., 2010; Singh and Singh, 2014). In terms of their ability to improve cheese flavor, strains of the *L. casei* group, especially the species *L. casei*, and *L. paracasei*, are one of the most extensively explored NSLAB. In Cheddar cheese, the application of *L. paracasei* strains as adjuncts improved both flavor intensity and bitterness (Lynch et al., 1999; Ong et al., 2007a). Milesi et al. (2010) showed that Cremoso cheeses with a *L. paracasei* adjunct strain had a similar composition to the control cheese with no adjunct, but the overall acceptability was higher. In Manchego cheeses, the addition of *L. paracasei* strains as adjuncts improved the flavor of the cheeses (Poveda et al., 2014). In Gouda cheeses, *L. paracasei* adjuncts contributed to cheese flavor diversification (Van Hoorde et al., 2010). Strain-specific effects of *L. paracasei* adjuncts originally isolated from Danbo cheese were observed when these strains were examined in a cheese model system, as some strains contributed to flavor improvement, while others led to the development of off-flavors (Antonsson et al., 2003).

In our previous work, we analyzed a set of *L. paracasei* NSLAB isolates to determine proteolytic characteristics in enzymatic assays, and their production of flavor compounds in cheese model systems (Stefanovic et al., 2017a,b). The objective of the present work was to determine whether the knowledge-based selected adjuncts contribute to cheese flavor diversification in short-aged Cheddar cheeses. To do this, the three strains showing significantly different activities in proteolytic enzyme assays, and in production of flavor compounds in cheese model systems were used as adjuncts in Cheddar cheese manufacture. Two of the strains (DPC4206 and DPC4536) shared Pulsed Field Gel Electrophoresis (PFGE) profiles, but demonstrated considerable differences in activities of proteolytic cascade enzymes, such as cell envelope proteinase, aminopeptidases, aminotransferase, and glutamate dehydrogenase (Stefanovic et al., 2017a). Additionally, the two strains differed in their ability to produce volatile flavor compounds in two cheese model systems (Stefanovic et al., 2017b). The third strain (DPC2071) had markedly different PFGE profile, showed high activity of cell envelope proteinase, and developed one of the most distinct volatile profiles in cheese model systems (Stefanovic et al., 2017a,b).

## Materials and methods

### Bacterial strains used in cheese manufacture

The starter culture used in cheese production was *Lactococcus lactis* ssp. *lactis* 303. In addition to the starter, three adjunct cultures, all belonging to the *L. casei* group, and designated as *Lactobacillus paracasei* (DPC2071, DPC4206, and DPC4536) were used. The strains were stored frozen at -80°C in the appropriate medium (LM17 broth (Merck, Darmstadt, Germany) for the starter culture, and MRS broth (Oxoid, Basingstoke, UK) for adjunct cultures, supplemented with glycerol [20% (v/v)]. Prior to the cheese making, strains were grown on LM17 or MRS agar plates, for starter and adjuncts, respectively, at 30°C.

### Cheese manufacture and ripening

Cheeses were manufactured at pilot scale, i.e., 500 L vats, at Moorepark Technology Ltd (Fermoy, Cork, Ireland). The Control cheese contained only starter culture, while each of the three test cheeses contained one of the three adjunct cultures in addition to the starter. Test cheeses were named according to the adjunct used (i.e., cheese DPC2071, cheese DPC4206, and cheese DPC4536). For cheese making, bulk starter cultures (1% v/v) were inoculated into 7 L of 10% (w/v) heat treated (90°C for 30 min) reconstituted skim milk (RSM) and incubated at 30°C for 18 h. After incubation, cultures were cooled, and kept at 4°C for 18 h until cheese manufacture the following day. Adjunct strains DPC2071 and DPC4206 were grown in 500 mL of 10% RSM (w/v) (autoclaved at 121°C for 5 min) containing addition of 1% (v/v) of yeast extract. Strain DPC4536 was grown in 500 mL of MRS broth, as previous tests showed its poor growth in milk (data not shown). For inoculation into the vat, the 500 mL culture of DPC4536 was centrifuged (4000 g, 10 min 4°C) and resuspended in 500 mL of sterile 10% (w/v) RSM. Three independent replicate cheese making trials were performed.

Raw milk was standardized to a protein-to-fat ratio of ~0.96:1. Milk was pasteurized at 72°C for 15 s and pumped into cylindrical, jacketed 500 L vats. Milk (454 kg/vat) was inoculated with starter and appropriate adjunct, as described above. Coagulation to obtain curd was achieved over 30 min by adding chymosin (18 mL/100 kg, Chr. Hansen, Cork, Ireland). After cutting, the curd and whey mixture was cooked at a rate of 1°C/5 min to a maximum scald of 38°C. Subsequently, the curds and whey were drained at pH 6.20. Curds were cut into smaller loaves, which were stacked on top of each other for additional draining, and regularly turned over, until pH of 5.3 was reached. Curds were milled and salted [2.75% NaCl (w/w)] and left to mellow for 20 min. Salted curds were molded (2 × 22 kg) and pressed for 18 h. Cheeses were vacuum-packed and ripened at 8°C for 3 months.

### Enumeration of starter and adjunct bacteria in cheeses

For bacteriological analysis, cheeses were aseptically sampled at Day 1, 14, 28, and Month 3 of ripening. The samples were placed in a sterile stomacher bag, diluted 1:10 with sterile 2% trisodium citrate and homogenized using a stomacher (Iul Instruments, Barcelona, Spain) for 5 min. Independent duplicate samples were taken at each time point and serial dilutions were prepared as required. Starter cells were enumerated on LM17 agar (Merck, Darmstadt, Germany) after incubation at 30°C for 3 days. Total NSLAB (lactobacilli) were enumerated on *Lactobacillus* selective (LBS) agar (BD, Oxford, UK) after 5 days incubation at 30°C. Coliforms and enterococci were enumerated on violet red bile agar (BD) and Kanamycin aesculin azide agar (Merck) after 24 h incubation at 30°C.

To confirm that the majority of NSLAB belonged to the inoculated adjuncts, Pulsed-Field Gel Electrophoresis (PFGE) was performed as described by Stefanovic et al. (2017a). Isolates from Month 3 samples were analyzed, and the PFGE patterns were compared with the patterns of the three adjuncts (Figure 2B).

### Cheese compositional and biochemical analysis

At Day 14 post manufacture, cheeses were sampled and grated for salt, protein, moisture, and fat content determination. Salt and moisture were determined according to the IDF methods (IDF (1979) and IDF (1982), respectively). Fat content was determined by CEM Smart Turbo Moisture/Solids analyser (CEM Corporation, Matthews, NC, USA). Additionally, primary and secondary proteolysis was monitored from Day 14 until Month 3 of ripening. Primary proteolysis was determined using the macro-Kjeldahl method (IDF, 1993). Secondary proteolysis was determined by measuring the free amino acid (FAA) content of the pH 4.6 soluble nitrogen extracts (pH4.6SN) according to the method described by McDermott et al. (2016). Samples of pH4.6SN were deproteinized by adding equal volume of 24% (w/v) tri-chloroacetic acid. After standing for 10 min, mixtures were centrifuged at 14,400 g (Microcentaur, MSE, UK) for 10 min. Supernatants were removed and diluted with 0.2 M sodium citrate buffer, pH 2.2 to give ~250 nmol/mL of each amino acid residue. Furthermore, samples were diluted (1:2 ratio) with norleucine as the internal standard, to give a final concentration of 125 nm/mL. Free amino acids were quantified using a Jeol JLC-500/V amino acid analyser (Jeol (UK) Ltd., Garden city, Herts, UK) fitted with a Jeol Na^+^ high performance cation exchange column.

### Free fatty acids (FFA) analysis of cheese lipid extracts by gas chromatography with flame ionization detector (GC-FID)

FFA content of cheeses was determined after 3 months of ripening. Lipid extraction was performed according to the procedure outlined by De Jong and Badings (1990) with the following modifications: 4 g of sample was mixed with 10 g anhydrous Na_2_SO_4_ by grinding with a mortar and pestle. One mL of an internal standard (ISTD) (C5, C11, C17 at 1,000 ppm in heptane) and 0.3 mL of 2.5 M H_2_SO_4_ were added to each sample. The samples were extracted 3 times with 15 mL of diethyl ether/heptane (1:1) and each time the solution was clarified by centrifugation at 3,000 g for 5 min. The collected extracts were pooled for solid phase extraction.

Aminopropyl columns (500 mg) were pre-conditioned with 10 mL of heptane. The lipid extract was applied to the column and the neutral lipids removed using 10 mL of 20% diethyl ether in hexane. At no point were the columns left to dry. FFA were collected using 5 mL of 2% formic acid/diethyl ether (2% FA/DE) in glass test tubes. The entire extract was immediately separated and stored in 2 mL amber vials which were capped with PTFE/white silicone septa (Agilent Technologies, Cork, Ireland). Amber vials were used to prevent ultraviolet light degradation of any polyunsaturated fatty acids that may be present in the extract.

Gas chromatography was performed on Varian CP3800 GC with a CP FFAP CB capillary column (30 m × 0.25 mm × 0.32 μm, Agilent Technologies) column in on-column mode. The injector was held at 25°C using cryogenics (liquid carbon dioxide) for 6 sec, this was raised to 250°C at 30°/min. The injector and auto-sampler were operated in on-column mode. The injection volume of the extracts obtained above was 0.5 μL. The inlet liner used was a SPI direct liner (Agilent Technologies). The carrier gas was helium and was held at a constant flow of 1.2 mL/min. The column oven was held at 40°C for 2 min and raised to 240°C at 7.5°C/min, and this was held for 23.33 min. The total runtime was 52 min. The Flame Ionization Detector (FID) was operated at 300°C. The identification of FFA in the samples was performed based on retention times of FFA in the standard mix (GLC Reference STANDARD 74 “Free acid,” Nu-Chek-prep, Inc. Waterville, MN, USA) used for the instrument calibration.

### Cheese volatile analysis by gas chromatography-mass spectrometry (GC-MS)

The volatile compounds present in the cheeses were determined after 3 months of ripening. For each cheese sample, 4 g of grated cheese was placed in an amber 20 mL screw capped Head-space Solid Phase (HS-SPME) vial with a silicone/PTFE septum (Apex Scientific, Maynooth, Ireland). Initially, the vials were equilibrated to 40°C for 10 min with pulsed agitation of 5 seconds at 500 rpm (Shimadzu AOC 5000 plus autosampler). The solid phase microextraction (SPME) was performed with a 50/30 μm Carboxen^TM^/ divinylbenzene/ polydimethylsiloxane (CAR/DVB/PDMS, Agilent Technologies, Cork, Ireland) fiber, which was exposed to the headspace above the samples for 20 min at 40°C. After extraction, the fiber was injected into the GC inlet and desorbed for 2 min at 250°C into a SPL injector with a SPME liner. Gas chromatography was performed on a Shimadzu 2010 Plus GC with a DB-5 (60 m × 0.25 mm × 0.25 μm, Agilent Technologies) column using a split/splitless injector in split mode 1:10. The carrier gas (helium) was maintained at 23 psi. The temperature of the column oven was set at 35°C, held for 5 min, increased at 6.5°C/min to 230°C then increased at 15°C/min to 320°C. The mass spectrometer detector Shimadzu TQ8030 was run in single quad mode. The ionization was done by electronic impact (−70 ev) and the mass range m/z scanned between 35 and 250 amu. All samples were analyzed in triplicate. To ensure there was no carry over between the samples, the SPME fiber was cleaned using a bake-out station at 270°C for 3 min. During the run, vials with external standards (dimethyl-sulfide, benzaldehyde, cyclohexanone, butyl acetate, acetone, and ethanol, all at concentrations of 10 ppm) were analyzed to ensure that analysis was done within specification. Blanks (empty vials) were injected regularly to monitor possible carry over.

The volatile compounds were identified using mass spectra comparisons to the NIST 2014 mass spectral library (Scientific Instrument Services, Ringoes, NJ, USA), Flavors and Fragrances of Natural and Synthetic Compounds Library and an in-house library created in GCMS Solutions software (Mason Technology) with target and qualifier ions and linear retention indices for each compound. Spectral deconvolution was also performed to confirm identification of compounds using AMDIS. An auto-tune of the GCMS was carried out prior to the analysis to ensure optimal GCMS performance. The compounds of interest were selected according to previously published review of compounds considered as main flavor contributors in cheese (Curioni and Bosset, 2002; Singh et al., 2003).

### Statistical analysis

To determine if significant differences exist in cheese composition, fat, salt, moisture, protein, FAA, and FFA content among cheese, samples were analyzed by Analysis of Variance (ANOVA) carried out using R statistical software (www.r-project.org/). Means were compared using the least significant difference (LSD) test, and the level of significance was determined at *p* < 0.05. For volatile analysis, the principal component analysis (PCA) was performed on selected signals resulting from chromatogram processing using the package FactomineR of the R software.

## Results

### Starter and adjunct enumeration showed typical evolution of cheese microbiota

**Figure 1:**
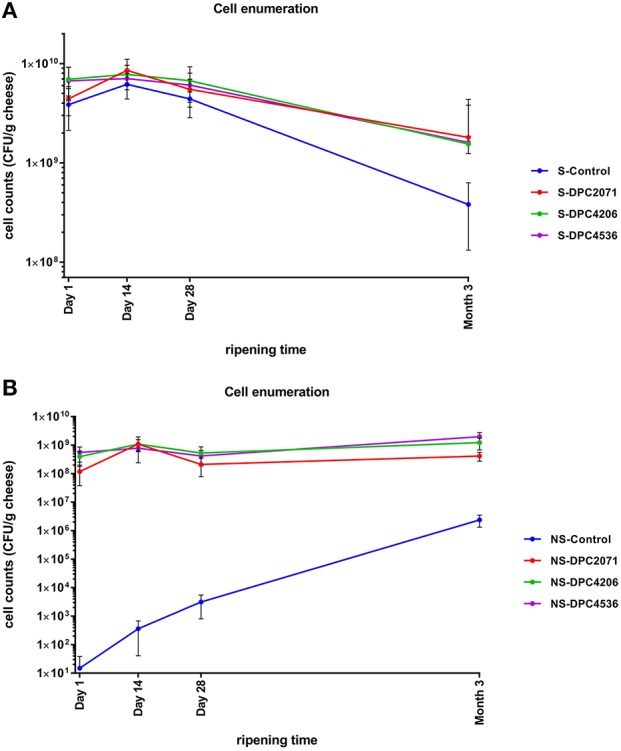
Enumeration of starter **(A)** and non-starter **(B)** lactic acid bacteria in cheeses during ripening. The values represent means obtained after enumeration of cells in cheeses of each of the three trials. Error bars represent standard deviation.

**Figure 2:**
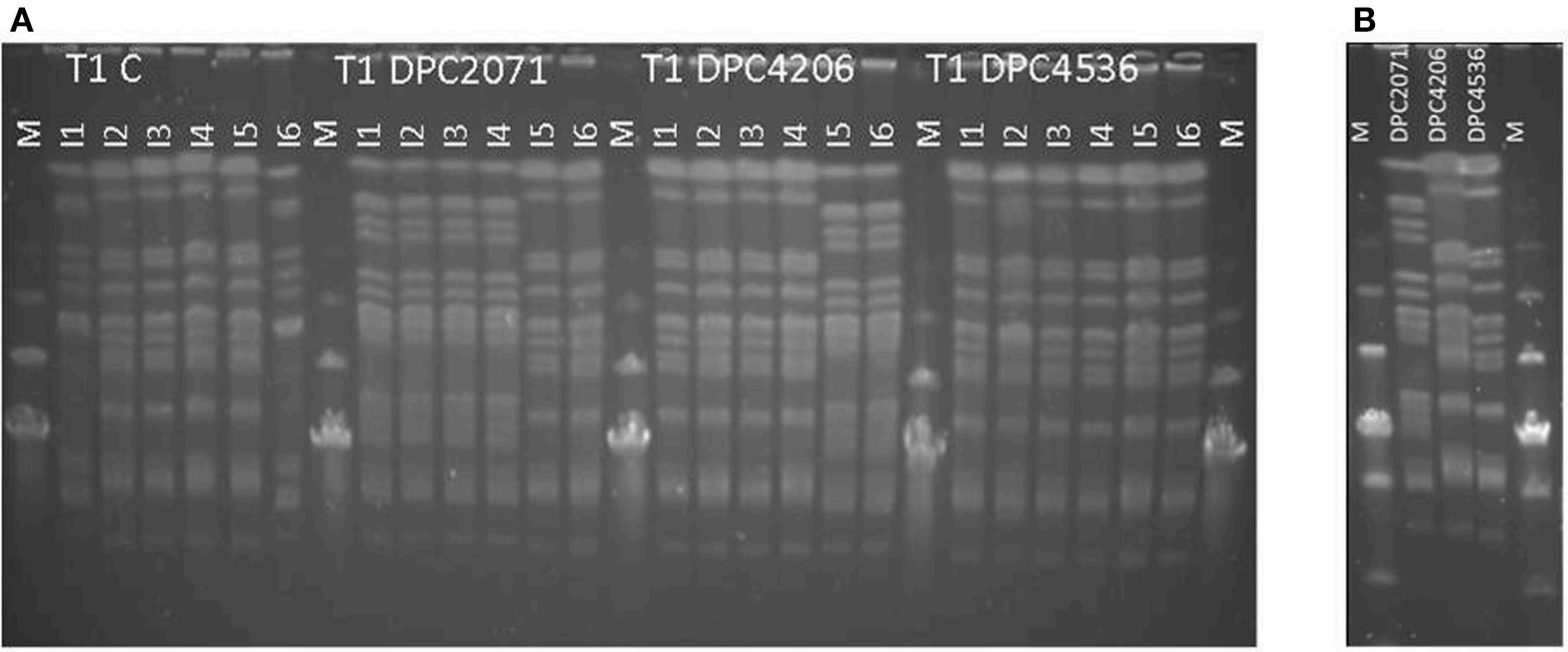
PFGE profiles of cheese (T1, Month 3 **(A)**). Six isolates (I1-I6) from the highest dilution obtained in cell enumerations in each cheese were evaluated. Figure represents results for isolates obtained from cheeses manufactured in trial 1 (T1). For comparison, in **(B)**, patterns of the three strains used as adjuncts are shown. M-Low range PFG marker, New England Biolabs.

In all four cheeses, similar trends in starter numbers were observed (Figure 1A). The starter culture was inoculated at ~4 × 10^9^-7 × 10^9^ CFU/g, and remained at similar levels in the first 1–28 days of ripening with a slight peak at Day 14. After 28 days of ripening, in all cases the numbers decreased by about 1–1.5 log_10_ units by Month 3, probably due to self-inhibition by acidification. Regarding the NSLAB counts, the test cheeses showed a similar trend, with a peak at Day 14 as occurred with the starter strain. At Day 28 levels decreased closer to the inoculation levels. At Month 3, levels increased again (Figure 1B). In the Control cheese, the numbers of NSLAB gradually increased over the ripening time from 10^1^ CFU/g at production to 10^6^ CFU/g at Month 3 of ripening. The use of PFGE profiles as an indicator of the presence of individual strains revealed that in each test cheese, at the end of ripening the patterns of NSLAB dominating at the highest dilution corresponded to the patterns of inoculated adjunct in each of the vats (Figure 2A). At Month 3, two out of the 6 (67%) of the strains isolated from cheese DPC2071 corresponded to strain DPC4206/DPC4536 pattern and vice versa, pointing to a putative cross-contamination. In Cheese DPC4536, all isolates corresponded to strain DPC4206/DPC4536 profile. In the Control cheese, various PFGE profiles were observed (Figure 2A), four of them corresponding to strain DPC4206/DPC5436 profile and the other two corresponding to unidentified strains. This finding may indicate a cross-contamination with strains DPC4206/DPC4536 and the development of adventitious NSLAB.

### The presence or choice of adjunct cultures did not influence gross cheese composition

The determination of cheese composition was performed on Day 14 (Table 1). No significant differences (*p* < 0.05) were shown between the cheeses in terms of fat, moisture, dry matter, salt, pH, salt in moisture (S/M), fat in dry matter (FDM), and moisture in non-fat solids (MNFS).

**Table 1:** The composition of manufactured cheeses at Day 14 of ripening.

**Compositional indices**	**Control cheese**	**DPC2071 cheese**	**DPC4206 cheese**	**DPC4536 cheese**
Moisture (%)	38.06	37.23	36.97	37.36
Salt (%)	1.79	1.76	1.79	1.78
pH	5.10	5.06	5.04	5.08
Fat (%)	29.72	30.19	30.36	30.24
Salt in moisture (%)	4.70	4.73	4.85	4.77
Fat in dry matter (%)	47.97	48.09	48.16	48.27
Moisture in non-fat solids (%)	54.14	53.33	53.08	53.55

### Free amino acid content in cheeses significantly differed in first weeks of ripening

**Figure 3:**
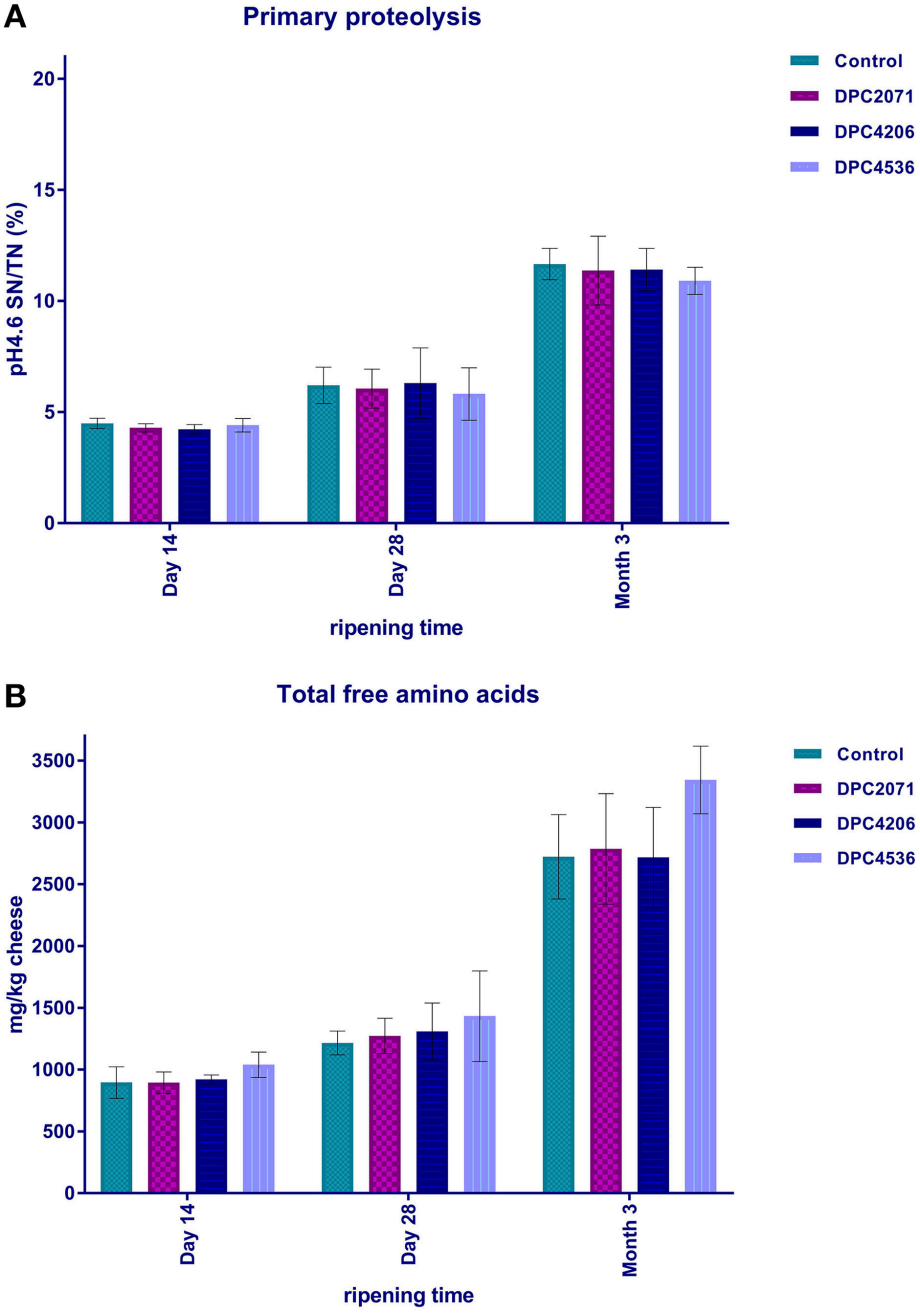
Primary proteolysis in cheese, expressed as the ratio of soluble nitrogen at 4.6 (WSN4.6) and total nitrogen **(A)**, and secondary proteolysis, expressed as mg of total free amino acids per kg of cheese **(B)**. Bars represent mean of three values. Error bars represent standard deviation.

The ratio between pH 4.6 soluble nitrogen and total nitrogen [pH4.6SN/TN (%)] was used to estimate the level of primary proteolysis. It increased significantly over ripening (*p* < 0.05) in all four cheeses, reaching ~11% at Month 3 (Figure 3A). No specific effect linked to the inoculated adjuncts was observed.

Secondary proteolysis was determined as the level of FAA liberated from peptides. A significant increase of total FAA at each of the cheeses was observed after Day 28 of ripening (*p* < 0.05, Figure 3B). When total FAA content was compared for the cheeses at each time point, no significant difference was observed (Figure 3B).

**Figure 4:**
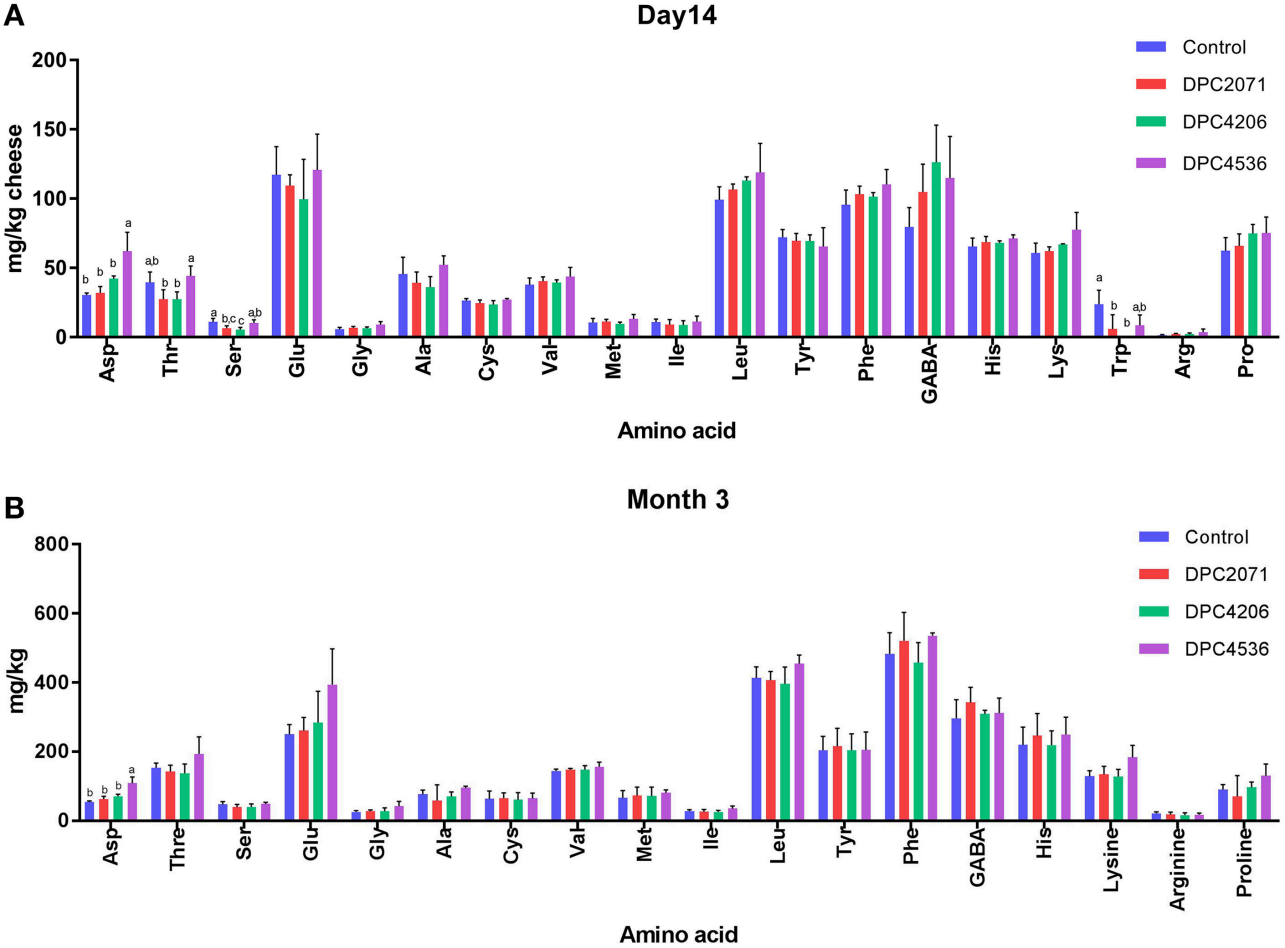
Free amino acids (mg/kg of cheese) present in two time points: **(A)** Day 14 of ripening and **(B)** Month 3 of ripening. Bars represent mean of three values. Error bars represent standard deviation. Letters (a,b) denote significant differences (*p* < 0.05) in abundances of free amino acids among four cheeses.

The concentrations of individual amino acids at each time point did not significantly differ among the four cheeses, except for aspartic acid, threonine, serine, and tryptophan in the samples at Day 14 (Figure 4A). No significant differences were observed at Day 28 among the samples, while at Month 3 significant difference was observed only in the case of aspartic acid, which was present at a significantly higher concentration in cheese DPC4536 (Figure 4B).

### The content of FFA in cheese lipid extracts did not significantly differ

There were no significant differences in the concentrations of any of 11 FFA analyzed in cheeses at Month 3. The concentration of C16 (palmitic) FFA was significantly higher in all cheeses compared to all other FFA. In addition, C18:1 (vaccenic), C18 (stearic), and C14 (myristic) were present in high concentration in all cheeses were but no significant differences in concentrations were observed (data not shown).

### Month 3 cheeses showed considerable differentiation in volatile profiles

After 3 months of ripening, 48 volatiles considered to contribute to cheese flavor were identified, 17 of which were present in significantly different (SD) abundances (Table 2). The ratio between the highest and the lowest value of abundances for a single compound among the four cheeses ranged between 1.85 for 3-methyl-3-buten-1-ol to >5000 for benzoic acid. The compounds present in SD abundances were present in higher abundances in test cheeses compared to the Control. The exceptions were butan-2-one, 3-methyl-buten-1-ol, and 3-methyl-buten-2-ol, the latter being in significantly higher abundances in the Control compared to the test cheeses. Cheese DPC4206 had the highest abundances of 3-methyl-buten-1-ol, 2-decenal, δ-dodecalactone, pentadecan-2-one, while cheese DPC4536 had the highest abundances of 2-decenal, δ-dodecalactone, pentadecan-2-one, and significantly higher abundances of ethyl octanoate, ethyl decanoate, octan-1-ol, benzoic acid and 2-undecenal compared to all other cheeses. Cheese DPC2071 was characterized by significantly lower abundances of octanal and 3-methyl-buten-2-ol compared to all other cheeses (LSD test, data not shown).

**Table 2:** Compounds identified in Cheddar cheeses after 3 months of ripening along with linear retention indices (LRI) used for compounds identification.

**Chemical group**		**LRI**		**Ratio**
**ALCOHOL**				
	Propan-1-ol	548	+	
	3-Methyl-3-buten-1-ol	728	+	1.85
	Pentan-1-ol	766	+	
	3-Methyl-2-buten-1-ol	772	+	2.46
	Octan-1-ol	1069	+	1028
**ALDEHYDE**				
	3-Methyl-butanal	654	+	
	Heptanal	903	+	
	Octanal	1003	+	668
	Benzeneacetaldehyde	1049	+	
	Nonanal	1106	+	2.54
	Decanal	1207	+	
	2-Decenal	1263	+	3416
	2-Undecenal	1365	+	3628
	Dodecanal	1410	+	
**KETONE**				
	2,3-Butanedione (Diacetyl)	591	+	
	Butan-2-one	598	+	6.49
	Pentan-2-one	684	+	
	2,3-Pentanedione	696	+	
	3-Hydroxy-butan-2-one (Acetoin)	732	+	
	Heptan-2-one	889	+	
	Nonan-2-one	1091	+	
	Pentadecan-2-one	1695	+	4774
**ACID**				
	Acetic acid	638	+	3.27
	Butanoic acid	792	+	
	Hexanoic acid	972	+	
	Benzoic acid	1155	+	5444
	Octanoic acid	1158	+	
	Decanoic acid	1353	+	5.39
**SULFUR COMPOUND**				
	Dimethyl sulfide (DMS)	518	+	
	Carbon disulfide (CDS)	537	+	
	Dimethyl disulfide (DMDS)	743	+	
	Dimethyl sulfone	921	+	
	Dimethyl trisulfide (DMTS)	979	+	
**ESTER**				
	Ethyl acetate	613	+	
	Ethyl butanoate	799	+	
	Ethyl hexanoate	996	+	
	Ethyl octanoate	1191	+	2.83
	δ-Octalactone	1289	+	2.18
	Ethyl decanoate	1387	+	5.52
	δ-Decalactone	1503	+	3.81
	γ-Dodecalactone	1685	+	
	δ-Dodecalactone	1716	+	3375
**OTHER**				
	Trichloromethane	623	+	
	2,5-Dimethylfuran	707	+	
	Toluene	769	+	
	m-Xylene	873	+	
	D-Limonene	1035	+	
	Undecane	1099	+	

*If the abundances of compound showed significant differences among cheeses (p < 0.05) the ratio between the maximal and minimal abundance of a compound between the cheeses was calculated. +: presence of a compound*.

**Figure 5:**
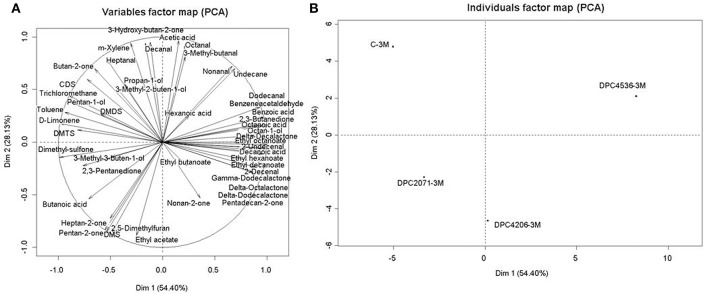
Individual factor map (**A)** and variable factor map **(B)** of principal component analysis (PCA) on 48 volatile compounds produced in Cheddar cheeses at Month 3 of ripening.

The PCA plot based on the abundances of all identified flavor contributors is presented in Figure 5. The first two axes described 82% of the total variability among cheeses, with dimension 1 (PC1) describing 54% of variability and dimension 2 (PC2) describing 28% of variability. Cheeses were discriminated mainly in PC1, while the Control cheese and cheese DPC2071 were discriminated in PC2. The position of the Control cheese was determined by butan-2-one, carbon-disulfide (CDS), 3-hydroxy-butan-2-one (acetoin), pentan-1-ol, dimethyl-disulfide (DMDS), dimethyl-trisulfide (DMTS), octanal, dimethyl-sulfone, decanal, propan-1-ol, 3-methyl-buten-2-ol, heptanal, acetic acid, and D-limonen. The position of cheese DPC2071 was determined by butanoic acid, ethyl butanoate, dimethyl-sulfone, and dimethyl-sulfide (DMS). Cheese DPC4206 was positioned according to the abundances of 2,3-pentanedione, DMS, hexanoic acid, ethyl acetate, nonan-2-one, heptan-2-one, 3-methyl-buten-1-ol, while the position of DPC4536 was determined by the abundance of benzoic acid, 3-methyl-butanal, octanoic acid, 2,3-butanedione, ethyl hexanoate, decanoic acid, γ- and δ- dodecanolactone, nonanal, undecane, dodecanal, and pentadecan-2-one (Figure 5).

## Discussion

In bacterial-ripened cheeses, such as Cheddar, the dynamic evolution of both the starter and non-starter microbial populations depends on environmental conditions and available nutrients during the manufacture and ripening thus affecting acidification, biochemical transformation of substrates and flavor development (Bautista-Gallego et al., 2014). Unlike the starter bacteria, whose viability is rapidly reduced in the initial phases of ripening, non-starter bacteria slowly increase in numbers to become the dominant microbiota in cheese (Gatti et al., 2014). In this study, cell enumerations of adjunct strains followed the general trends observed during Cheddar cheese ripening (Fox et al., 1998; Sousa et al., 2001; Settanni and Moschetti, 2010). The starter bacteria grew during cheese manufacture and were maintained at high numbers during the early phase of ripening (Day 28), after which starter numbers decreased, probably due to the utilization of most of the lactose (Crow et al., 2002) and the inhibitory effect of salt and low pH (Chapman and Sharpe, 1981). The most abrupt decrease of starter occurred in the Control cheese, probably pointing to a protective role of the adjuncts over the starter strain in the cheeses inoculated with adjunct. Conversely, the numbers of adjuncts inoculated in the three test cheeses maintained high levels over ripening. In the Control cheese, with no adjunct added, a typical evolution of NSLAB microbiota was observed (Fox et al., 1998; Steele et al., 2006). NSLAB numbers started increasing after Day 28 and at Month 3 reached level of 10^6^ CFU/g, which is a considerable level but probably not high enough to induce a major effect on flavor development.

The three strains used as adjunct cultures were previously isolated as part of the non-starter microbiota of different Cheddar cheeses produced in Ireland. Each of the three strains was previously designated as *Lactobacillus paracasei*, according to the results of sequencing of 16S rRNA PCR amplicons (Stefanovic et al., 2017a).

To evaluate the persistence and the dominance of the added adjuncts in relation to naturally-present or contaminating flora in the cheese, PFGE profiles in Month 3 cheeses were obtained. The PFGE patterns showed that the dominating profiles corresponded to the inoculated *L. paracasei* strains in each vat (Figure 2A). In the Control cheese, PFGE patterns of adjuncts used in three tested cheeses as well as patterns not representing the adjunct strains were detected. The presence of tested adjuncts points to cross-contamination which can occur in the setting of the pilot plant where cheeses were manufactured (open vats and simultaneous production of all four cheeses). Patterns not matching to used adjuncts presumably corresponded to other contaminating microbiota originating from the environment, personnel or the pasteurized milk.

The gross composition of the manufactured cheeses was determined at Day 14. No significant difference in any of the parameters was observed between the four cheeses, indicating that inoculated adjuncts had no effect on the cheese composition. Gilles and Lawrence (1973) proposed a grading system of cheese quality according to the values of cheese composition indices, such as salt in moisture (S/M), fat in dry matter (FDM), moisture in non-fat solids (MNFS), and pH. In terms of the overall quality of the cheeses produced in this study, the only parameter deviating from “premium grade” is FDM, which in our case is ~48%. In the case of premium grade cheeses FDM values are typically between 52 and 55%, while values of 50–56% are typical in lower graded cheeses. Since lipolysis in Cheddar cheese is not extensive, the fat content plays a minor role in determining cheese quality, and FDM values from a relatively wide range are acceptable. However, if FDM value is lower than 48%, it is highly possible that cheese will be firmer and have less acceptable flavor at the end of ripening (Fox et al., 2004). Taking into account all of the parameters, cheeses produced in this study were of satisfactory quality. In addition, texture analysis was carried out through the whole ripening and no differences were observed among cheeses in any of the ripening points (data not shown).

Primary proteolysis in cheese refers to the degradation of casein into large polypeptides, mainly driven by chymosin (rennet) in the initial phases of ripening (Gobbetti et al., 2007) and by plasmin, although the latter is of less significance due to the pH of the cheese. The level of primary proteolysis can be expressed through various calculations (McSweeney and Fox, 1997) and the most common way is the ratio between soluble nitrogen in cheese extracts at a pH of 4.6 and total nitrogen (pH4.6SN/TN). In this study, the levels of pH4.6SN/TN significantly increased during ripening, and the results obtained correspond to typical trends observed in Cheddar (Lane and Fox, 1996). However, no significant differences among the cheeses were observed in terms of primary proteolysis (Figure 3A). These results confirm that adjunct (or NSLAB) bacteria have a minimal effect on primary proteolysis in cheese, as shown by Lane and Fox (1996). In cheeses where *L. paracasei* strains are added primarily for their probiotic effect, they showed only a minor impact on proteolysis (Bergamini et al., 2006). Similarly, Bielecka and Cichosz (2017) showed that addition of *L. paracasei* LPC-37 during ripening of a Dutch-type cheese did not significantly affect proteolysis and peptidolysis. In contrast, Ong et al. (2007b) showed that after 20 weeks of ripening, Cheddar cheeses with added *L. casei* and *L. paracasei* had significantly higher values of water-soluble nitrogen (WSN) compared to the control cheese and cheeses with other probiotic adjuncts. This confirms the contribution of added *L. paracasei* strains to proteolysis, whether used as flavor adjuncts or probiotics, is strain-specific. In the present study, no such differences were observed. However, if we had decided to manufacture different type of cheese, ripened under different conditions, and if different starter culture(s) was used to initiate acidification, selected adjuncts could have performed better and contributed to bigger differentiation in levels of proteolysis. The degradation of large polypeptides to shorter peptides and FAA by proteinases and peptidases is considered as secondary proteolysis in cheese (Gobbetti et al., 2007). As expected, the level of total FAA in the cheeses increased over ripening time. However, statistical differences in the concentrations of individual amino acids among the cheeses were observed only at the early stage of ripening (Day 14). Similar results for control and adjunct-added cheeses were reported by Lane and Fox (1996), where it was shown that the peptidase systems of added lactobacilli contributed much less to the release of FAA than starter peptidases, that are capable of degrading a wide range of medium and small peptides to amino acids. Although the adjunct strains were selected for this study on the basis of variable enzymatic activities and their contribution to proteolysis (Stefanovic et al., 2017a), we did not observe any direct or synergistic effect of the inoculated adjuncts toward secondary proteolysis. A direct correlation between the concentration of FAA and cheese flavor cannot be established, since different types of cheeses have similar relative proportions of amino acids, but differ distinctly in flavor (Sousa et al., 2001). In addition, as metabolism of amino acids seems to be strain-specific, a similar pool of FAA will be converted to different volatiles by different strains (Peralta et al., 2016). In this study, a limited differentiation of cheeses was observed based on FAA levels. This was also reflected in GC volatile profiles of the cheeses, where significant differences in concentrations of some FAA-derived volatiles were observed. The examples are benzoic acid, originating from phenylalanine, that has rosy, honey-like aroma, and branched-chain alcohols, 3-methyl-buten-1-ol and 3-methyl-buten-2-ol, that originated from leucine, and are known for their cheese, fruity notes (Curioni and Bosset, 2002).

Cheddar cheese belongs to the group of cheeses with moderate levels of lipolysis (Collins et al., 2003). In Cheddar cheese, starter cultures are observed as the main FFA producers, while the contribution of NSLAB appears to be minimal (Hickey et al., 2006). Free fatty acids (C4-C12), lactones and methyl-ketones are important flavor contributors with low threshold points (Collins et al., 2003; O'Mahony et al., 2005). The lipolytic activity of both starter and adjunct cultures were confirmed in a quantitative assay with 4-nitrophenyl-dodecanoate (data not shown), although the network of lipolytic reactions occurring in cheese is much more complex and involves numerous specific and non-specific enzymes. The analysis of FFA content at Month 3 confirmed the minimal contribution of adjunct cultures to lipolysis in Cheddar cheese. As expected, palmitic acid was present in the highest levels of all FFA across all the cheeses, followed by stearic and vaccenic acids, since it is known that C16 and C18 acids dominate in bovine milk triglycerides (Collins et al., 2003).

The differences observed in the volatile profiles of the analyzed cheeses occurred, apart from several FAA-derived compounds (reported above), mainly from lipolysis-driven compounds. Since no significant differences in FFA contents were observed, it can be implied that the main lipolytic reactions arose from starter activity, but the further development of flavor-contributing compounds came from the adjunct metabolism of some of the primarily developed metabolites. This metabolic activity was dominant in cheese DPC4536 and partially in cheese DPC4206. Numerous long chain aldehydes, acids, and lactones were present in the volatile profile of cheese DPC4536, and to a lesser extent in cheese DPC4206 and the Control cheese. Octanal and nonanal have green, fatty aroma (Curioni and Bosset, 2002) while 2-decenal and 2-undecenal are characterized by green grass-like and herbaceous aromas (Verzera et al., 2004; Ziino et al., 2005). Decanoic acid has stale butter flavor (Curioni and Bosset, 2002). Lactones, such as δ-octalactone, δ-decalactone, and δ-dodecalactone mainly contribute to the coconut, fruity notes, similarly as ethyl esters (octanoate, decanoate) (Curioni and Bosset, 2002). This differentiation of cheeses based on volatile profiles was confirmed in PCA plot. The Control cheese was separated from all test cheeses, showing that adjuncts did contribute to differentiation in volatile profiles of cheeses. Additionally, test cheeses were separated from each other as well, showing that overall volatile profiles were different depending on the adjunct used. Apart from benzoic acid, the main variables leading to the differentiation were the aforementioned lipid metabolites. However, although the ratio between the highest and the lowest abundance detected in cheeses for some compounds was considerably high (>2000), this was a consequence of the complete absence of these volatiles in the profiles of some cheeses, and their presence in the profiles of other cheeses. For example, benzoic acid was detected in the Control cheese and cheeses DPC4206 and DPC4536, but was completely absent in cheese DPC2071, which contributed to the ratio of >5000.These compounds were, in a statistical sense, a factor of differentiation; however, their realistic contribution is not just based on abundance but odor activity. The previous characterization of strains that were used as adjuncts showed considerable differences in enzyme activities and in volatile profiles obtained in cheese model systems. This was the initial groundwork for our knowledge-based approach in adjunct selection. The selected adjuncts (DPC2071, DPC4206, and DPC4536) showed differences in enzymes of proteolytic cascade and generated diverse volatile profiles in cheese model systems. In this study, we showed that in short-aged Cheddar cheese, these adjuncts were able to contribute to development of different volatile profiles, implying different flavor characteristics. However, although some differentiation in volatile profiles occurred based on differences in volatiles arising in FAA metabolism, surprisingly, the major impact on differentiation was by lipid metabolism derived compounds. The reason for this could be that the increase in complexity of the environment may have caused minimization of adjunct metabolic differences and smaller impact of protein-derived compounds. On the other hand, the availability of lipids and starter-driven lipid metabolites and potentially favorable conditions during ripening contributed to generation of variable abundances of lipid metabolites, thus affecting overall diversity of volatile profiles of cheeses.

In addition to all reported results, the ripening of the manufactured cheeses was maintained until total of 9 months of ripening. During this time, cell enumeration, primary, and secondary proteolysis was determined and volatile analysis at Month 9 was performed. These results are not reported, since they showed that no significant differences occurred in any of the determined parameters during the prolonged ripening. In addition, PCA analysis of volatiles in Month 9 showed that cheeses had more similar profiles among themselves compared to ones obtained in Month 3. The sensorial analysis (Ranking descriptive analysis) that was performed after 9 months of ripening confirmed a high degree of similarity among the cheeses, since scores for only three out of 20 analyzed attributes were significantly different (data not shown). The main issue we encountered during prolonged ripening was the dominance of the three adjunct strains in the Control cheese, observed in PFGE profiles at Month 9. For this reason, in late stage of ripening, the Control cheese did not represent a true experimental control. The development of NSLAB microbiota in the Control cheese that corresponded to adjuncts inoculated in the test cheeses most likely diminished differences that would potentially be observed if the cross contamination did not occur. This led to decrease in differentiation between the Control cheese and three test cheeses at the end of the prolonged ripening, compared to differentiation observed at Month 3, where the Control cheese was still not predominantly populated by the adjuncts, and total number of NSLAB was low. However, it cannot be ruled out that these adjuncts could be successfully used in flavor diversification in long-ripened Cheddar cheese.

In this study, the influence of three adjunct *Lactobacillus paracasei* strains in flavor development of short-aged Cheddar cheese was assessed. The adjunct strains did not show an impact on gross composition, nor did they influence primary or secondary proteolysis or lipolysis. Volatile analysis at Month 3 showed that the differences in volatile profiles among the four cheeses were caused mainly by the variation in long-chain aldehydes, acids and esters that originated from the metabolism of FFA. On the other hand, flavor compounds originating from FAA metabolism showed limited variation, although strains were selected based on their previously determined proteolytic activity. This study confirmed that adjuncts of *L. paracasei* are able to contribute to versatile flavor compounds production in short-aged Cheddar cheese.

## Authors contributions

ES and CR performed cheese trials and all laboratory work. All authors contributed to the design of the study and writing the manuscript.

### Conflict of interest statement

The authors declare that the research was conducted in the absence of any commercial or financial relationships that could be construed as a potential conflict of interest.
